# Interactions of *Echinacea* spp. Root Extracts and Alkylamides With the Endocannabinoid System and Peripheral Inflammatory Pain

**DOI:** 10.3389/fphar.2021.651292

**Published:** 2021-04-27

**Authors:** Rui Liu, Nadia L. Caram-Salas, Wei Li, Lili Wang, John Thor Arnason, Cory Steven Harris

**Affiliations:** ^1^ Department of Biology, University of Ottawa, Ottawa, ON, Canada; ^2^ Departamento de Innovacion Biomédica, Unidad de Desarrollo y Évaluacion Preclinica de Sustancias Bioactivas, Catédra CONACYT-CICESE, Ensenada, Baja California, Mexico; ^3^ Beijing Institute of Pharmacology and Toxicology, Beijing, China

**Keywords:** echinacea, purple coneflowers, cannabinoid receptor agonists, inflammation, peripheral pain, alkylamides

## Abstract

Historical ethnobotanies of indigenous peoples of the North American prairies reveal treatment of many painful conditions by *Echinacea* spp. Recent evidence suggests a pharmacological basis for such use as the bioactivity of *E. angustifolia* and *E. purpurea* is mediated, in part, through activation of the endocannabinoid system (ECS). Whereas the cannabimimetic effects of individual echinacea products and alkylamides have been described, the activity of crude extracts has not been compared between cannabinoid (CB) receptors or across species or genotypes. Moreover, few studies have explored echinacea’s engagement of the ECS for historic treatments or new therapeutic applications in peripheral inflammatory pain. We hypothesized that 1) the *in vitro* effects of root extracts on CB receptor internalization would vary with species and phytochemistry, and that echinacea root extracts would reduce inflammatory pain *in vivo* through activation of the ECS. Root extracts of different *E. angustifolia* and *E. purpurea* accessions were prepared, analyzed by HPLC-DAD to quantify caffeic acid derivatives and alkylamides (AKA), and tested for agonist and antagonist activities using receptor redistribution assays. Linear regression of activity relative to phytochemistry identified predictive compounds that were assessed individually in redistribution assays. Extracts were evaluated in the Hargreaves model of chronic inflammatory pain in rats with co-administration of selective CB1/2 antagonists to gauge involvement of the ECS. CB receptor agonist activity varied among accessions of both species with linear regression revealing a significant relationship between CB1 activity and AKA2 for *E angustifolia*, and AKA 9 + 10 for *E purpurea*. CB2 activity was positively related with AKA 9 + 10 and total AKAs in *E. angustifolia*. Four isolated AKA demonstrated agonist activity in the CB2, but not CB1, assay. In the inflammatory pain model, oral administration of either *E angustifolia* or *E. purpurea* root extract produced dose-dependent analgesic effects that were partially reversed by co-administration of CB receptor antagonists. This study demonstrates that *in vitro* effects of crude echinacea root extracts on CB receptors is predicted by phytochemistry. *In vivo,* echinacea has potential applications for peripheral inflammatory pain such as arthritis and burns, reflecting the traditional uses of Indigenous North Americans.

## Introduction

Echinacea is perhaps the best known medicinal plant of North America and has a long and rich cultural history of use. Classic ethnopharmacology research on echinacea, mostly with *Echinacea purpurea* (L.) Moench and *E. angustifolia* DC (Asteraceae), has focused mainly on activities such as antimicrobial action and immunomodulation in relation to traditional pharmacopoeial uses for colds and flu (Catanzaro et al., 2018). These uses find their origin in the practices of 19th century Eclectic physicians who borrowed knowledge of indigenous peoples of the prairies (Great Plains) of North America.

In addition to these familiar uses, there is an extensive ethnobotanical record of other uses of *Echinacea* spp. uses (Moerman, 1998; Binns, 2001). These reports show that *Echinacea* spp. were also used extensively by indigenous cultures for management of pain, for example tooth ache by the Niitsitapi (Blackfoot First Nation), arthritis by the Tsestho’e (Cheyenne tribes) and rheumatism or burns by the Šakówiŋ (Dakota and Lakota First Nations). Recent research has revealed a relevant new mechanism of pain management by echinacea mediated by alkylamides (AKA) acting at the cannabinoid (CB) receptors (Woelkart et al., 2005; Raduner et al., 2006; Hohmann et al., 2011). In addition to selectively binding and activating CB2 receptors, certain echinacea alkylamides (AKA) can modulate endocannabinoid system (ECS) activity through effects on endocannabinoid metabolism and transport (Chicca et al., 2009; Rui et al., 2020). Among other physiological and pathophysiological functions, the ECS plays a key role in regulating inflammatory pain (Nagarkatti et al., 2009), acute pain states (Alkaitis et al., 2010) and nociceptive pathways in chronic pain (Guindon and Hohmann, 2009; Rahn and Hohmann, 2009; Guindon and Hohmann, 2011; Rani Sagar et al., 2012), highlighting the role of endocannabinoids as endogenous analgesics.

While the cannabimimetic activity of pure alkylamides has been well-described in experimental models, particularly in the context of inflammation, the activity of crude extracts – and how it varies with species and phytochemistry – remains poorly studied. Moreover, despite the ethnopharmacological evidence, research into echinacea’s activity in models of peripheral pain is surprisingly limited. Accordingly, the present study investigated a collection of phytochemically characterized *E. angustifolia* and *E. purpurea* root extracts in CB1 and CB2 receptor assays, predicting that activity would vary with AKA content and that regression analysis would identify phytochemicals predictive of activity for future breeding purposes. Based on the observed *in vitro* activities of extracts from both species, two pooled extracts of *E. angustifolia* and *E. purpurea,* respectively, were studied in a well-established animal model of arthritic peripheral inflammatory pain. Activity was compared to two positive controls (dexamethasone, diclofenac) and the role of the ECS was investigated with CB1 and CB2 antagonists.

## Materials and Methods

### Plant Materials and Extraction

A selection of *E. purpurea* (n = 9) and *E. angustifolia* (n = 11) genotypes were selected and cloned by plant breeders John Baker, Phil Hintz and co-author Arnason in a previous study of germplasm grown at Trout Lake Farms WA. The root samples were dried at 45°C and milled to powder (1 mm mesh). Each powdered sample (500 mg) was extracted three times in 15 ml fresh 70% ethanol using ultrasound (5 min) followed by centrifugation (10 min, 3200 rcf) and collection of the supernatant. Supernatants were dried under vacuum (Speedvac) followed by lyophilization (SuperModulyo220 freeze dryer; Thermo fisher scientific, Nepean, ON, Canada).

### Phytochemical Analysis

An Agilent HPLC system (model 1100) with a Phenomenex Luna (C18, 100 × 2.1 mm, 5 um particle size; Phenomenex Inc. Mississauga, Ontario) column was used for phytochemical analysis. Detailed methods for identification and quantification of targeted compounds, as well as the purification of AKAs, were described previously (Liu, 2019). Analytical standards of caffeic acid derivatives were obtained from Sigma-Aldrich. All solvent used in HPLC and UPLC/MS analysis were optima LC/MS grade solvent purchased from Fisher scientific.

### CB Receptor Redistribution Assay

Stock solutions of root extracts, alkylamides, and positive controls for CB receptor agonism (Win55,212-2, Toronto Research Chemical, Toronto) and antagonism (SR141716, Sigma-Aldrich, Oakville) were prepared by dissolving corresponding compounds/extracts in ethanol and subsequently diluting them with medium to final concentrations of 600 and 2500 μg/ml for plant extracts; 4, 12, 40, and 120 μg/ml for AKAs; 0.4 and 4 μm for Win55,212-2 and 4 μm for SR141716.

Green fluorescent protein-tagged CB1 (CB1-GFP) and CB2 (CB2-GFP) fusion protein expressed U2OS cell lines were obtained from Thermo Fisher Scientific (Beijing, China) and the assay procedure followed the CB 1/2 Redistribution Assay protocol from Thermo Fisher. In brief, cells were cultured in DMEM (high glucose) medium with 0.5 mg/ml G418 and 10% FBS in a 96 well plate contain DMEM F12 medium with 10 mm HEPES, 1% FBS and 1 μm Hoechst (33342) at a concentration of 8,000 cells/100μl/well with 5% CO2 at 37°C for 18–24 h.

For the agonist assay, each well was first washed with 100 μl of medium followed by adding 150 μl of medium and 50 μl of pre-diluted samples or positive control. Cells were then incubated under 5% CO2 at 37°C for 120 min Win55,212-2 (final concentration at 1 μm) was used as positive control and 0.5% ethanol was used as vehicle control.

For the antagonist assay, each well was first washed with 100 μl of medium followed by adding 100 μl of medium and 50 μl of per-diluted samples and positive control. Cells were then incubated under 5% CO2 at 37°C for 60 min followed by adding 50 μl medium containing 0.4 μm Win55,212-2. Cells were then incubated for 120 min under the same conditions. Rimonabant (final concentration = 1 μm) was used as positive control and 0.5% ethanol was used as vehicle control.

IN Cell Analyzer 1000 Cellular Imaging and Analysis System (GE Healthcare Bio-Sciences Corp) was used to monitor the CB receptor internalization. The excitation/emission was set at 350/460 nm for Hoechst 33342 and 475/535 nm for GFP with 300 and 500 ms exposure time respectively. The magnification was set at ×20 objectives and 5 photos in different regions of each well were taken. The qualitative analysis of GFP spot formation was done by using an IN-Cell Analyzer 1000 Granularity Analysis Module.

The average of 15 images from triplicate wells per sample was used to calculate the percentage activation or inhibition. The following formulae were used to calculate the % activity (% activation or % inhibition) of echinacea root extracts and AKAs on CB receptors:

$$\text{\% Activity }=\;\left[1\;-\frac{{(\text{Endosomes GFP}}_{\text{Positive control}}{\;-\text{ Endosomes GFP}}_{\text{sample}})}{{(\text{Endosomes GFP}}_{\text{Positive control}}{\;-\text{ Endosomes GFP}}_{\text{vehicle}})}\right]\times 100\text{\%}$$

$$\text{\% Inhibition }=\frac{{(\text{Endosomes GFP}}_{\text{Positive control}}{\;-\text{ Endosomes GFP}}_{\text{sample}})}{{(\text{Endosomes GFP}}_{\text{Positive control}}\;-\text{ Endosomes GFPvehicle})}\text{ × }100\text{\%}$$

### Animals

Complete Freund’s Adjuvant (CFA; heat-killer *M. Tuberculosis*) and Dexamethasone were purchased by Sigma-Aldrich, St. Louis, MO, United States. Female Wistar rats (180–220 g body weight) obtained from the animal facility of CICESE were used in this study. Rats were housed (six per cage) in acrylic cages (44 cm width × 33 cm length × 20 cm height) with free access to drinking water, but food was withdrawn 8 h before experiments. Rats were placed in a controlled temperature (22 ± 1°C) and a controlled light inverted cycle (12-h light/12-h dark) (lights off at 7:00 h) room. All experiments followed the Guidelines on Ethical Standards for Investigation of Experimental Pain in Animals (Zimmermann, 1983) and the Mexican regulation (NOM-062-ZOO-1999) and were carried out according to a protocol approved by the local Animal Ethics Committee. The number of experimental animals was kept to the minimum needed to observe significant effects. All compounds and extracts were dissolved in saline and administered orally.

### Hargreaves Inflammatory Pain Model

Peripheral inflammatory pain was evaluated using the Hargreaves model of regional polyarthritis. Inflammatory pain was induced by injecting a low volume (100 µl) of Complete Freund’s Adjuvant (CFA; heat-killed *M. tuberculosis,* Sigma-Aldrich, St. Louis, MO) suspended in oil: saline 1:1 emulsion into the right hind paw. The paw withdrawal latency in response to the application of a radiant stimulus onto the plantar surface of both right and left paw was measured using the plantar Analgesia Meter equipment for paw stimulation (IITC Life Science, Woodland Hills, CA, United States) as described in Farrington et al., 2014. The time taken by the animal to respond by licking or flicking its paw was interpreted as positive response (paw withdrawal latency). All animals that presented a baseline response below 15 s prior to the injection of CFA were excluded from the study. A cutoff time (20 s) was established at the end of which the heat source shut off automatically to avoid tissue damage. Animals were kept (randomized) 1 per cage.

We used six animals per group (n = 6) to minimize the biological variability. Rats received vehicle (saline) or increasing doses of *E. angustifolia* (2.5–10 mg/kg), *E. purpurea* (2.5–10 mg/kg), diclofenac (30 mg/kg, Sigma-Aldrich) or dexamethasone (4 mg/kg, Sigma-Aldrich) by oral administration. The analgesic effect was measured each 30 min over 7 h. All experimental results are expressed as the mean ± SEM for six animals per group. Curves were constructed by plotting the latency of the paw withdrawal as a function of time. An increase of the latency was considered as analgesic effect. Area under the curve (AUC) was calculated by the trapezoidal method to obtain the % of Maximum Possible Effect (%MPE) as a representation of % of analgesia.

The %MPE was calculated as:

$$\text{\% MPE }=\;\frac{{(\text{AUC }}_{\mathrm{Compound}}{\;-\text{ AUC }}_{\text{CFA}}{\;}_{\text{injected paw}})}{{(\text{AUC }}_{\text{Normal paw}}{\;-\text{ AUC }}_{\text{CFA}}{\;}_{\text{injected paw}})}\text{× }100\text{\%}$$

### Statistical Analysis

For CB receptor agonist and antagonist activity *in vitro* as well as effects on paw withdrawal in the chronic inflammatory pain model, a one-way ANOVA followed by Dunnett’s test relative to vehicle control were used to analyze the data. Simple linear regression modelling was used to investigate potential relationship between CB1/2 receptor agonist activity and log transformed concentration of major components in echinacea root extracts. Prism GraphPad (v.7.0) was used to conduct all analyses.

## Results

### Phytochemical Analysis of Echinacea Root Extracts

**FIGURE 1:**
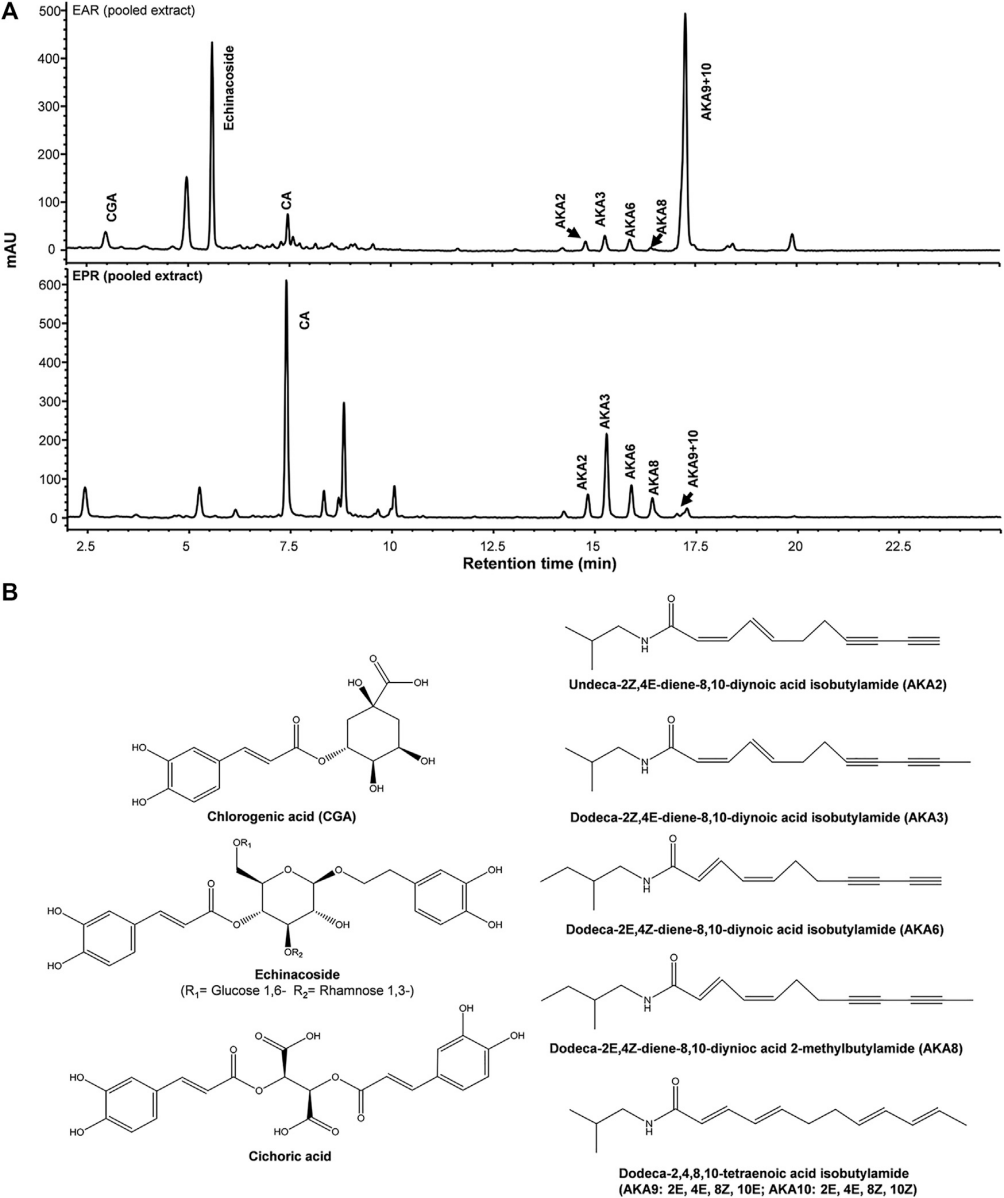
Representative HPLC-DAD chromatograms of pooled *E. purpurea* (EPR) and *E. angustifolia* root extracts (EAR) at 280 nm **(A)**. Chemical structures of major caffeic acid detivatives and alkylamides (AKA) **(B)**.

Major components from *Echinacea* spp. breeding accessions were quantified by HPLC-DAD (Figure 1). Echinacoside was the major caffeic acid derivative (CAD) found in the root extracts of *E. angustifolia* while cichoric acid was the dominant CAD found in *E. purpurea*. Dodeca-2E,4E,8Z,10Z/E-tetraenoic acid isobutylamide (AKA9/10) were the dominant AKA in *E. angustifolia* root extract, accounting for at least 60% of the total AKA content, and no AKA8 was found in the tested *E. angustifolia* root extracts. The major AKAs in *E. purpurea* root extracts were more evenly distributed as no single AKA exceeded more than 30% of the total AKA content. Variation of major components was also observed between accessions of the same *Echinacea* spp., up to 12-fold for different individual components (Table 1), providing a range of concentrations and profiles to identify those that contribute to, or are predictive of, ECS activity.

**TABLE 1:** HPLC-DAD quantification of major components in *Echinacea spp* root extracts. Mean concentration in each genotype (technical replicates, n = 3) of caffeic acid derivatives [chlorogenic acid (CGA), echinacoside and cichoric acid (CA)] and alkylamides (AKA) are presented together with mean concentration across coded genotypes of each species. Coefficients of variation for technical replicates were below 8% of mean values. Pooled extracts used for animal trials differed from means due to varying yields among pooled root extracts.

*Code*	*% Yield*	*Concentration in extract (µg/mg)*							
		CGA	Echinacoside	CA	AKA2	AKA3	AKA6	AKA8	AKA9+10
*EAR01*	21.5	0.93	18.25	0.95	0.79	1.42	1.14	N/A	14.46
*EAR07*	36.4	0.91	8.32	0.42	1.30	0.45	0.22	N/A	12.85
*EAR09*	19.9	1.82	19.89	3.45	1.30	2.86	1.35	N/A	17.91
*EAR13*	38.1	5.59	5.87	3.06	5.28	1.41	0.67	N/A	13.84
*EAR15*	31.5	3.45	21.78	2.94	0.41	0.92	0.50	N/A	2.77
*EAR22*	19.0	3.23	8.34	3.21	1.97	1.27	0.65	N/A	6.37
*EAR23*	42.1	1.38	15.12	0.78	0.23	0.82	0.39	N/A	15.01
*EAR24*	42.9	1.96	15.58	1.71	0.35	1.19	0.73	N/A	5.88
*EAR25*	22.6	1.98	36.69	2.57	0.25	1.30	0.70	N/A	8.84
*EAR35*	30.9	2.75	6.64	1.10	3.13	1.65	1.22	N/A	15.48
*EAR39*	40.9	2.90	23.64	1.83	0.34	1.07	0.49	N/A	5.72
Pooled extracts		1.48 ± 0.01	27.38 ± 0.31	1.06 ± 0.03	0.46 ± 0.01	0.75 ± 0.01	0.54 ± 0.01	N/A	7.17 ± 0.14
*EPR01*	19.0	N/A	N/A	42.84	3.60	9.06	4.41	5.56	1.57
*EPR02*	23.4	N/A	N/A	12.04	8.18	18.09	11.62	5.57	1.92
*EPR05*	13.8	N/A	N/A	23.73	13.55	18.51	12.84	9.79	6.02
*EPR07*	20.6	N/A	N/A	44.02	6.41	19.77	10.04	12.93	4.17
*EPR27*	25.0	N/A	N/A	12.42	6.14	10.48	7.23	5.29	2.43
*EPR31*	21.9	N/A	N/A	25.49	7.03	13.67	7.42	5.91	5.24
*EPR32*	32.6	N/A	N/A	19.86	5.66	7.15	5.47	5.11	3.89
*EPR36*	17.9	N/A	N/A	71.07	15.69	28.60	12.36	8.85	3.98
*EPR50*	28.4	N/A	N/A	6.21	3.01	12.59	4.84	4.40	2.51
Pooled extracts		N/A	N/A	32.05 ± 0.22	3.92 ± 0.07	13.22 ± 0.07	5.17 ± 0.05	4.35 ± 0.07	1.64 ± 0.18

* Pooled extracts used in reported *in vivo* experiments.

### CB Receptor Internalization

**FIGURE 2:**
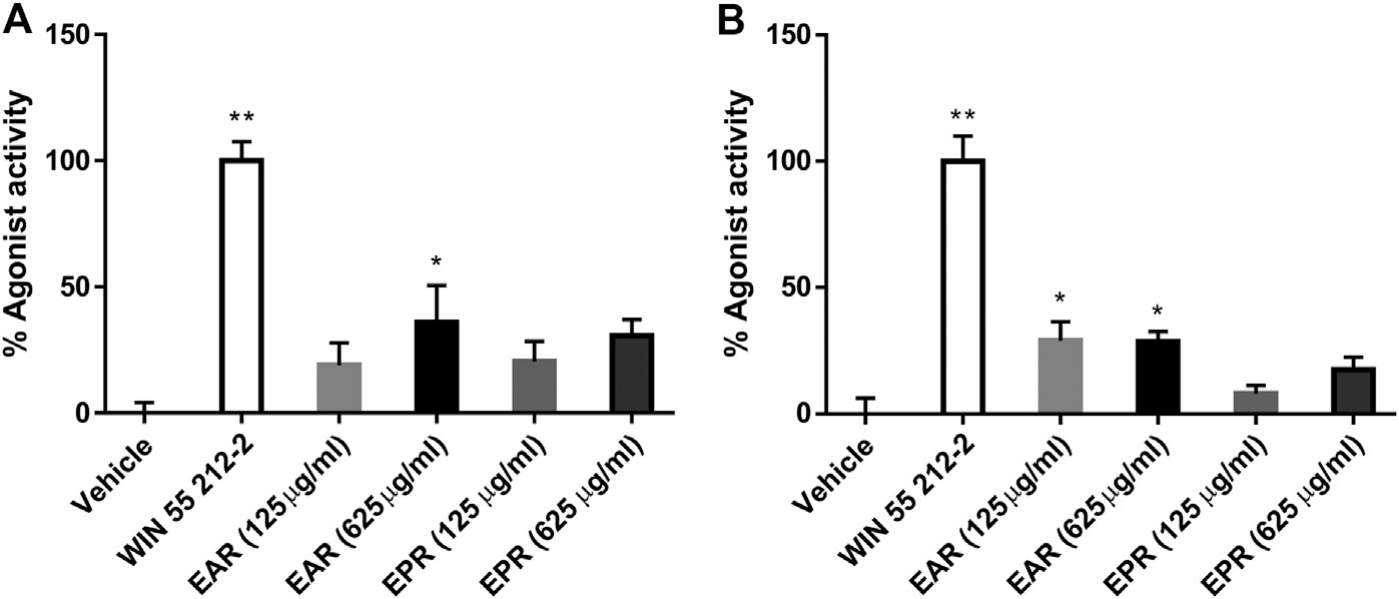
Mean agonist effects of *E. angustifolia* (EAR, *n* = 11) and *E. purpurea* (EPR, n = 9) root extracts on CB1 **(A)** and CB2 **(B)** receptor in CB receptor redistribution assays, expressed relative to the positive control WIN 55,212-2 (1 µm). An analysis of variance (ANOVA) was performed to evaluate the variance between samples followed by Dunnett’s test *post hoc* relative to the vehicle (0.5% ethanol) control. *p < 0.05; **p < 0.01.

The CB receptor redistribution assays quantify the receptor internalization following activation (i.e. exposure to agonist). It is not a functional assay of CB-receptor signaling but is often used as a measure of agonist and antagonist activity (Daigle et al., 2008; van der Lee et al., 2009) and, given the established CB receptor binding and activation by certain echinacea AKAs, the assay served as an indicator of extract and AKA agonist (or antagonist) effects. Eleven *E. angustifolia* and nine *E. purpurea* root extracts representing different accessions were evaluated at two concentrations for agonist activity at CB1 (Figure 2A) and CB2 (Figure 2B). Mean agonist activity of *E. angustifolia* accessions (n = 11) was significant at both receptors, with *E. purpurea* extracts eliciting weaker effects (on average, n = 9). Notably, a one-way ANOVA comparing accessions within each species revealed significant variation in observed agonist activity at both receptors, with some extracts eliciting no effects while others acted comparably with the positive control (1 µm Win55,212-2).Whereas only a few *E. angustifolia* and *E purpurea* root extracts showed weak antagonist effects when tested in the presence WIN55212-2, most extracts of both species tended to increase the effects of the co-administered agonist. Across *E. angustifolia* extracts, receptor internalization was significantly elevated compared to WIN55212-2 at both CB1 and CB2. As observed in agonist assays, *E. purpurea* extracts showed a similar yet weaker activity profile (Supplementary Material S1).

**FIGURE 3:**
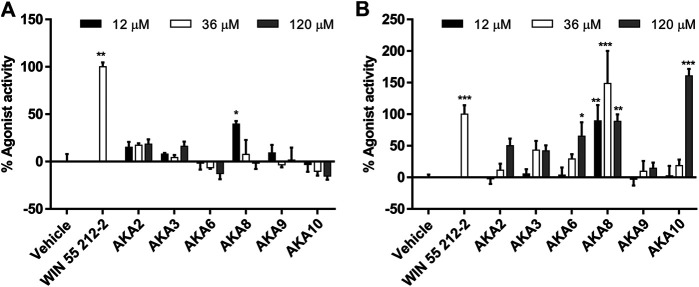
Mean agonist effects of isolated Echinacea alkylamides (AKA) on CB1 **(A)** and CB2 **(B)** receptor in CB receptor redistribution assays, expressed relative to the positive control WIN 55,212-2 (1 µm). An analysis of variance (ANOVA) was performed to evaluate the variance between samples followed by Dunnett’s test *post hoc* relative to the vehicle (0.5% ethanol) control. *p < 0.05; **p < 0.01; #*p* < 0.001.

Isolated AKAs from echinacea extracts were tested in the same agonist assays; most compounds were only weak agonists or inactive in the CB1 assay (Figure 3A), and only AKA8 displayed significant activity at 3 μg/ml. However, the agonist effect of most of the AKAs on CB2 were concentration-dependent and significant (*p* < 0.05) compared to control. An exception was AKA 9, an isomer of 10, which was inactive (Figure 3B). In the presence of WIN55212-2, no compounds elicited antagonist effects at CB1 but several AKAs (2, 3, 6 & 8) significantly increased receptor internalization. No significant antagonist or sensitization activity was observed at CB2 in the presence of WIN55212-2 (Supplementary Material S1).

### Linear Regression Analysis of Activity of Accessions With Their Phytochemical Content

**FIGURE 4:**
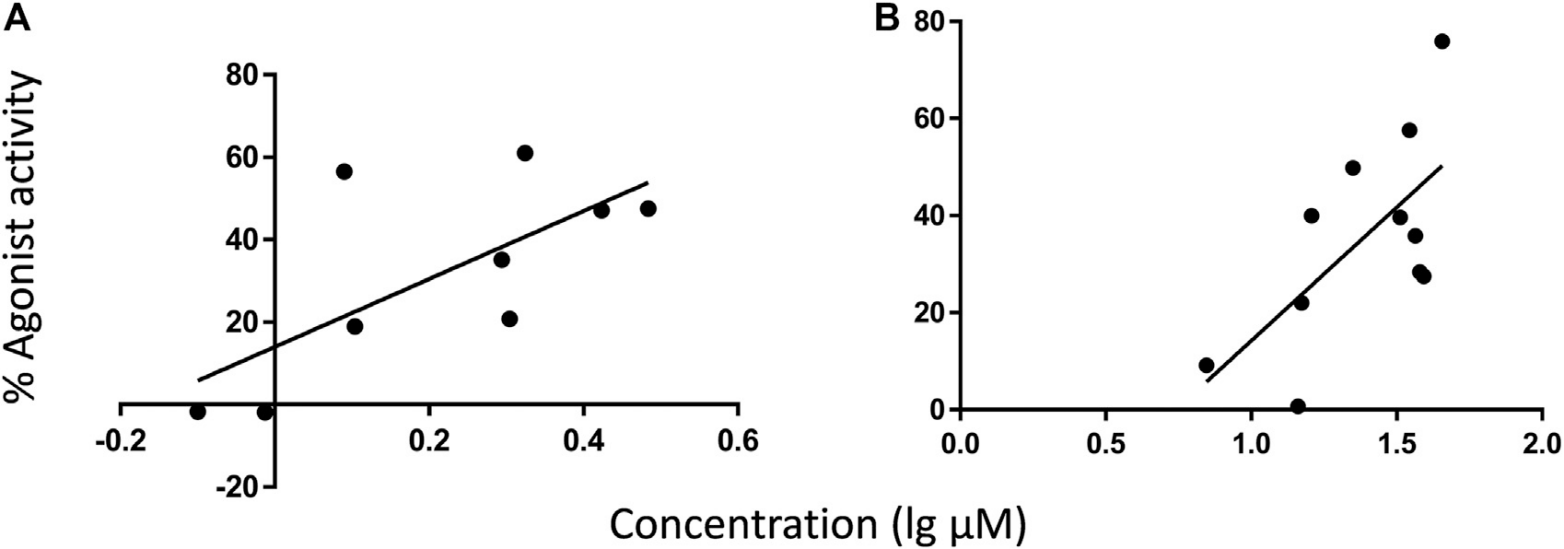
Regressions of CB receptor internalization (% agonist activity) relative to AKA9 + 10 concentration in different root extracts under experimental conditions. AKA9 + 10 concentrations were positively related to agonist activity at CB1 among *E. purpurea* accessions **(A)**, and at CB2 among *E. angustifolia* accessions **(B)**. AKA concentrations listed in Table 1 were adjusted to in-well concentration then converted from µg/ml to µm (MW of AKA9/10 = 247.38 g/mol). Refer to Table 2 for linear regression results.

Simple linear regression of CB1 agonist effects relative to individual and total CAD or AKA in 11 *E. angustifolia* root extracts revealed a significant positive relationship with AKA2 that explained 37% of variation in activity in the low concentration group (Table 2). Among nine *E purpurea* root extracts, the combined concentration of AKA9 + 10 was detected as the significant independent variable for agonist effects, explaining 48% of variation (Figure 4A). No other component, nor total AKA content, was significantly related to CB1 activity (Table 2). For CB2, regression of agonist activity relative to the measured concentrations of major components across *E. angustifolia* extracts showed that 43% of variation was significantly predicted by AKA9 + 10 concentration in the crude extracts (Figure 4B). Further analysis suggested that total AKA content improved the amount of variation explained to 51% (Table 2). CB2 agonist activity did not correlate with any identified *E. purpurea* phytochemicals.

**TABLE 2:** Simple linear regression of CB1 receptor agonist effects as a function of log-concentration of major phytochemical components of the 11 genotypes of *E. angustifolia* and 9 genotypes of *E. purpurea* extract. Results are shown for total extract concentration of 0.125 mg/ml for CB1 and 0.625 mg/ml for CB2. CAD, caffeic acid derivatives; AKA, alkylamide.

	*E. angustifolia*			*E. pupurea*		
CB1	Slope	*R* ^2^	*P*	Slope	*R* ^2^	*P*
Echinacoside	−57.91	0.22	0.15	N/A	N/A	N/A
Chicoric acid	−24.57	0.05	0.49	5.29	<0.01	0.85
AKA2	40.86	**0.37**	0.05*	18.37	0.09	0.37
AKA3	−22.77	0.02	0.67	6.39	<0.01	0.9
AKA6	−10.31	0.01	0.82	30.53	0.05	0.55
AKA8	N/A	N/A	N/A	71.62	0.23	0.19
AKA9+10	30.48	0.06	0.47	82.34	**0.48**	0.04*
Total CAD	−81.87	0.24	0.12	5.29	<0.01	0.85
otal AKA	43.44	0.09	0.36	37.47	0.07	0.49
**CB2**						
Echinacoside	−14.25	0.03	0.62	N/A	N/A	N/A
Chicoric acid	17.37	0.06	0.57	−11.83	0.05	0.55
AKA2	21.68	0.23	0.14	44.99	0.25	0.17
AKA3	50.91	0.23	0.14	−4.26	<0.01	0.88
AKA6	32.5	0.13	0.28	26.11	0.08	0.46
AKA8	N/A	N/A	N/A	38.68	0.13	0.34
AKA9+10	54.93	**0.43**	0.03*	3.86	<0.01	0.91
Total CAD	−7.56	<0.01	0.84	−11.83	0.05	0.55
Total AKA	67.6	**0.51**	0.01*	31.58	0.1	0.41

Significant relationships (*p* < 0.05) are indicated in bold.

### Rat Chronic Inflammatory Pain Model

**FIGURE 5:**
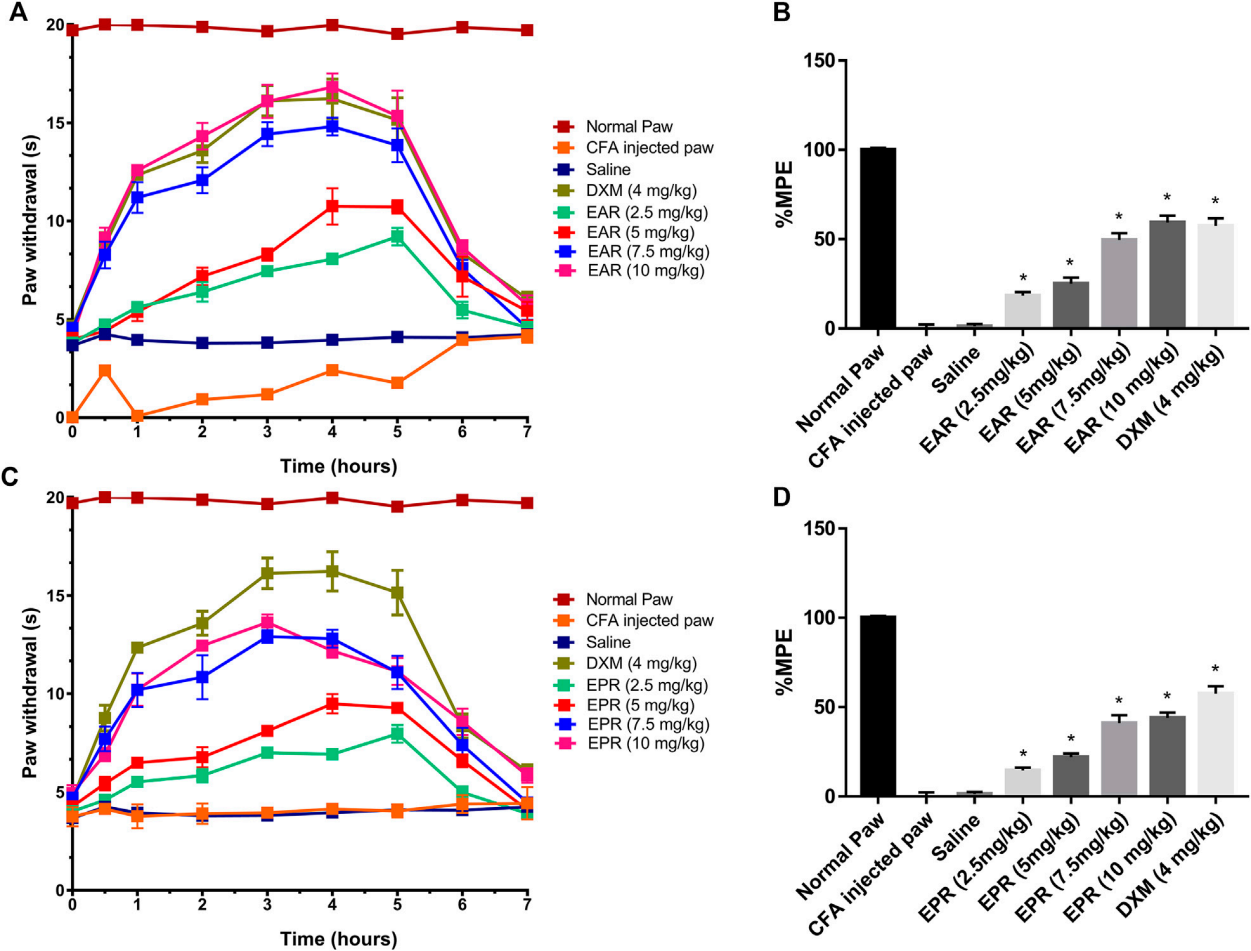
Time course of paw withdrawal responses **(A, C)** observed after acute oral administration of *E. angustifolia* root extract (EAR) or *E. purpurea* root extract (EPR) and dexamethasone in peripheral pain in rats and corresponding area under the curve (AUC) for each treatment expressed as percent of maximum possible effect (%MPE) **(B, D)**. The Normal Paw group is placed as a reference of the maximum possible effect. Saline solution was used as vehicle control for Complete Freund’s Adjuvant (CFA). In all cases, data are presented as mean ± SEM for 6 rats. Significant differences were determined by ANOVA analysis and *post hoc* Dunnett’s t-test relative to saline vehicle (*p < 0.01).

To follow up on the observed *in vitro* activity of *Echinacea* ssp. in the CB receptor redistribution assays, pooled extracts of *E. angustifolia* roots (EAR) and *E. purpurea* roots (EPR) were tested separately in the Hargreaves chronic inflammatory pain assay, an experimental model for polyarthritis. Oral administration of EAR (Figures 5A,B) and EPR (Figures 5C,D) induced a dose dependent (2.5–10 mg/kg) inhibition of thermal hyperalgesia in the rat. At 10 mg/kg, EAR induced 60% reversal of thermal hyperalgesia with an overall response duration of 5 h (Figure 5A). Similarly, the administration of EPR induced a dose-dependent analgesic effect reversing up to 50% of thermal hyperalgesia for 5 h (Figure 5C). Both echinacea extracts at the highest dose (10 mg/kg) provided similar results to the positive drug control group treated with dexamethasone (4 mg/kg) over the 7-h testing period.

**FIGURE 6:**
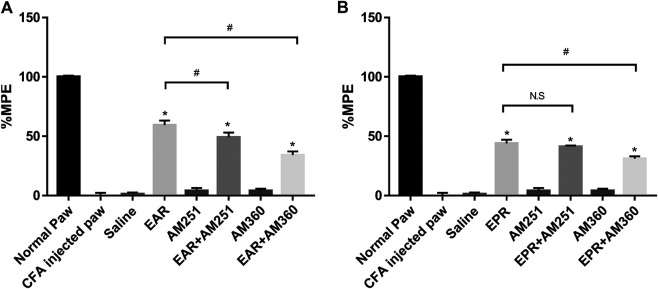
Effect of the oral administration of **(A)** *E. angustifolia* root extract (EAR) or **(B)** *E. purpurea* root extract (EPR) and cannabinoid receptor antagonists AM251 (CB1) and AM630 (CB2) on peripheral pain in rats. Data are presented as % of maximum possible effect (%MPE), relative to the Normal Paw group, as a function of dose. Data are the mean ± SEM for 6 animals. * Significantly different from the vehicle (Saline) group (*p* < 0.01); #Significantly different from the EAR without antagonist group (*p* < 0.01). as determined by ANOVA analysis followed by *post hoc* Dunnett’s t-test. N.S: not significant (*p* > 0.05).

To investigate whether either echinacea extract suppressed inflammatory pain/thermal hyperalgesia through the ECS, CB receptor antagonists AM251 (CB1) and AM630 (CB2) were administered in combination with echinacea extracts in the Hargreaves model. The results indicated that the suppression of thermal hyperalgesia by EAR was significantly reduced when co-administrated with either AM251 or AM630 (Figure 6A). However, pharmacological response of EPR was only significantly reduced by the CB2 antagonist AM630 but not the CB1 antagonist AM251 (Figure 6B). In addition, both CB receptor antagonists alone showed no activity in this animal model at the tested concentrations (Figure 6).

## Discussion

While the activity of certain AKAs and *Echinacea* spp. extracts in CB receptor assays has been reported previously, this is the first report of activity in the CB redistribution assay, which follows receptor internalization as a measure of agonist or antagonist activity, and the first comparison of breeding accessions to identify phytochemical predictors of activity. Our results reconfirm the agonist activity of specific AKAs and extracts in this new assay and, as previously reported (Woelkart et al., 2005; Raduner et al., 2006), are selective for CB2. Regressions of activity relative to phytochemical characteristics of the accessions revealed significant positive relationships with AKA9 + 10 isomers (dodeca-2E,4E,8Z,10E/Z-tetraenoic acid isobutylamide) as well as total AKAs (Table 2; Figure 4). The CB-receptor binding and signaling activity of AKA10 (Raduner et al., 2006) and the isomer pair (Woelkart et al., 2005) have been well described but this is the first study to test both AKA9 and 10 separately and demonstrate strong stereo-selective activity of the 10Z isomer (AKA10) at CB2. The weaker agonist effects observed among *E. purpurea* accessions likely reflect the lower concentrations of AKA9 + 10 in these extracts, despite the higher concentrations of other AKAs (Table 1). Cichoric acid does not bind to either CB1 or CB2 (Raduner et al., 2006) and, based on our regression results, receptor internalization was unrelated to individual or total CADs, indicating that these metabolites are not interacting directly with CB receptors. Whereas greater statistical power (i.e. a larger collection of genotypes) would improve capacity to identify actives/markers and potential synergistic effects, our data show that selection for higher AKA10 levels in *E. angustifolia* and *E. purpurea* breeding programs should increase ECS activity.

In CB receptor antagonist assays, the agonist effects of crude extracts and certain AKAs appeared to be additive to the effects of WIN55212-2, leading to negative % antagonist activities (i.e. more internalization than agonist alone, Supplementary Material). Our unexpected observation that several AKAs that lacked CB1/2 agonist activity enhanced receptor internalization in the presence of WIN 55,212-2 deserves further investigation. Chicca et al (2009) similarly reported that AKAs can enhance 2-AG induced signaling ([Ca^2+^] transients) in HL60 cells and anandamide transport in U937 cells via micellation. Given the lack of supplemented carrier protein (e.g. albumin) in assay medium, AKAs may have facilitated extracellular transport of lipophilic WIN55212-2.

The ECS targets of echinacea components, especially AKAs, are not limited to CB receptors and transporters but also include endocannabinoid degradating enzymes. Certain, but not all, echinacea extracts inhibit the Fatty acid amide hydrolase (FAAH) in *in vitro* assays (Chicca et al., 2009), with both CAD and AKA contributing to activity (Liu et al., 2020). FAAH inhibitors have been reported to effectively reduce pain in rodent models of osteoarthritis by reducing inflammatory flares (McDougall et al., 2017). Furthermore, alkylamides are known to be metabolized by hepatic CYP450 enzymes following oral administration. Two studies have evaluated the impact of hepatic metabolism on AKA activities; Cech et al. (2006) reported reduced suppression of IL-2 secretion in stimulated T cells by echinacea alkylamides (AKA9 + 10) after hepatic oxidation. Liu (2019), in contrast, observed enhanced FAAH inhibition of AKA following *in vitro* metabolism using human liver microsomes (Liu 2019). Together, current evidence suggests the overall pharmacological outcome of orally consumed echinacea products is not determined by a single active ingredient but a variety of components and their metabolites, which can interact with the ECS through different mechanisms.

For purposes of breeding and product development, this study is the first to assay germplasm accessions to determine if selection for high activity genotypes is feasible. High and low activity samples were clearly differentiated based *in vitro* assays, especially for CB2 receptor agonism, and this may be a practical way to select germplasm for targeted development as a treatment for managing pain and inflammation, as demonstrated in the animal model.

A very promising result is the demonstration that both echinacea root extracts have dose dependent activity in the rat paw model of chronic inflammatory pain. In fact, the results show that the extract is just as effective as dexamethasone (at concentrations 2–3 times higher than this corticosteroid pharmaceutical), a remarkable level of activity for a crude plant extract. Future comparisons with other analgesics, particularly THC (or cannabis extracts), would inform echinacea’s potential as an alternative treatment for inflammatory pain. Although the treatment outcome was positive, the link to pharmacological mechanism and active principles *in vivo* remains incomplete. The overall anti-inflammatory effect of *Echinacea* spp extracts are considered to be the net effect of several classes of compounds. The anti-inflammatory potential of AKAs, CADs and essential oils have all been reported, acting through various mechanism *in vivo* following oral administration (Chao et al., 2009; He et al., 2009; Yu et al., 2013; Manayi et al., 2015; Zhu et al., 2015). AKAs, however, are the most bio-available components from echinacea and, in addition to established ECS-mediated anti-inflammatory potential, can reportedly inhibit cyclooxygenase-1 and -2 (Clifford et al., 2002) as well as NF-κB expression (Matthias et al., 2008).

The connection between the ECS and inflammation, particularly inflammatory pain, is well established (Nagarkatti et al., 2009) as both CB1 and CB2 agonists provide effective treatment *in vivo* (Clayton et al., 2002). Our results are the first to demonstrate the analgesic potential of echinacea in a chronic inflammatory pain model. Suppression of inflammatory pain was partially blocked by CB2 antagonist AM630 for both species, and by CB1 antagonist AM251 for EAR, data that reflect the relative agonist effects observed using receptor internalization assays (Figures 2, 3). Whereas CB2 antagonism reduced the effects of both EAR and EPR extracts to a similar degree, activation of CB1 appears to account for the stronger analgesic response to EAR relative to EPR. Accordingly, while activation of peripheral CB2 receptors contributed strongly to the analgesic effects of both extracts (likely by reducing inflammation), *E. angustifolia* may also act on central CB1 receptors and offer greater therapeutic potential. Interestingly, as reported previously for echinacea’s anti-inflammatory activity (Gerstch, 2006), analgesic mechanisms were not limited to the ECS as both extracts elicited significant responses in the presence of CB antagonists. Whether this non-ECS response is mediated by anti-inflammatory or anti-nociceptive mechanisms warrants further investigation. Ongoing work has already begun to assess the anti-inflammatory effect of isolated alkylamides in this chronic inflammatory pain model.

Ethnobotanical sources showed that Echinacea spp. were traditionally used by First Nations as treatment for a variety of painful conditions including sore throat, burns, arthritis, tonsillitis and wounds, among other conditions and symptoms (Moerman, 1998). The present study suggests a pharmacological basis for these uses and a method for germplasm selection. Our animal model is relevant to suggest it may be active in chronic pain conditions appropriate for NHP or dietary supplement use, such as arthritis, carpel tunnel syndrome etc. In these conditions, inflammation and pain are symptoms that may respond well to *E. angustifolia* treatments. Although *Echinacea* spp. extacts *per se* have not been investigated clinically, the effect and safety of a highly standardized ginger (*Zingiber officinale*) plus echinacea (*Echinacea angustifolia*) extract supplementation on inflammation and chronic pain in NSAID poor-responders has shown efficacy in a pilot study in human subjects with knee arthrosis (Rondanelli et al., 2017). Other neuropathic pain conditions such as diabetic and chemotherapy-induced neuropathies could be investigated and clinical evaluation of *Echinacea spp.* extracts or formulations for some of these conditions is warranted as an alternative or complementary treatment.

## Data Availability

The raw data supporting the conclusions of this article will be made available by the authors, without undue reservation.
