# Supplementary material for: Interactions of Echinacea spp. Root Extracts and Alkylamides With the Endocannabinoid System and Peripheral Inflammatory Pain
Source: Front Pharmacol. 2021 Apr 27;12:651292. doi: 10.3389/fphar.2021.651292 (PMC8111300; doi:10.3389/fphar.2021.651292)
Supplement: Supplementary file 1 [file image1.pdf]

Supplemental Material

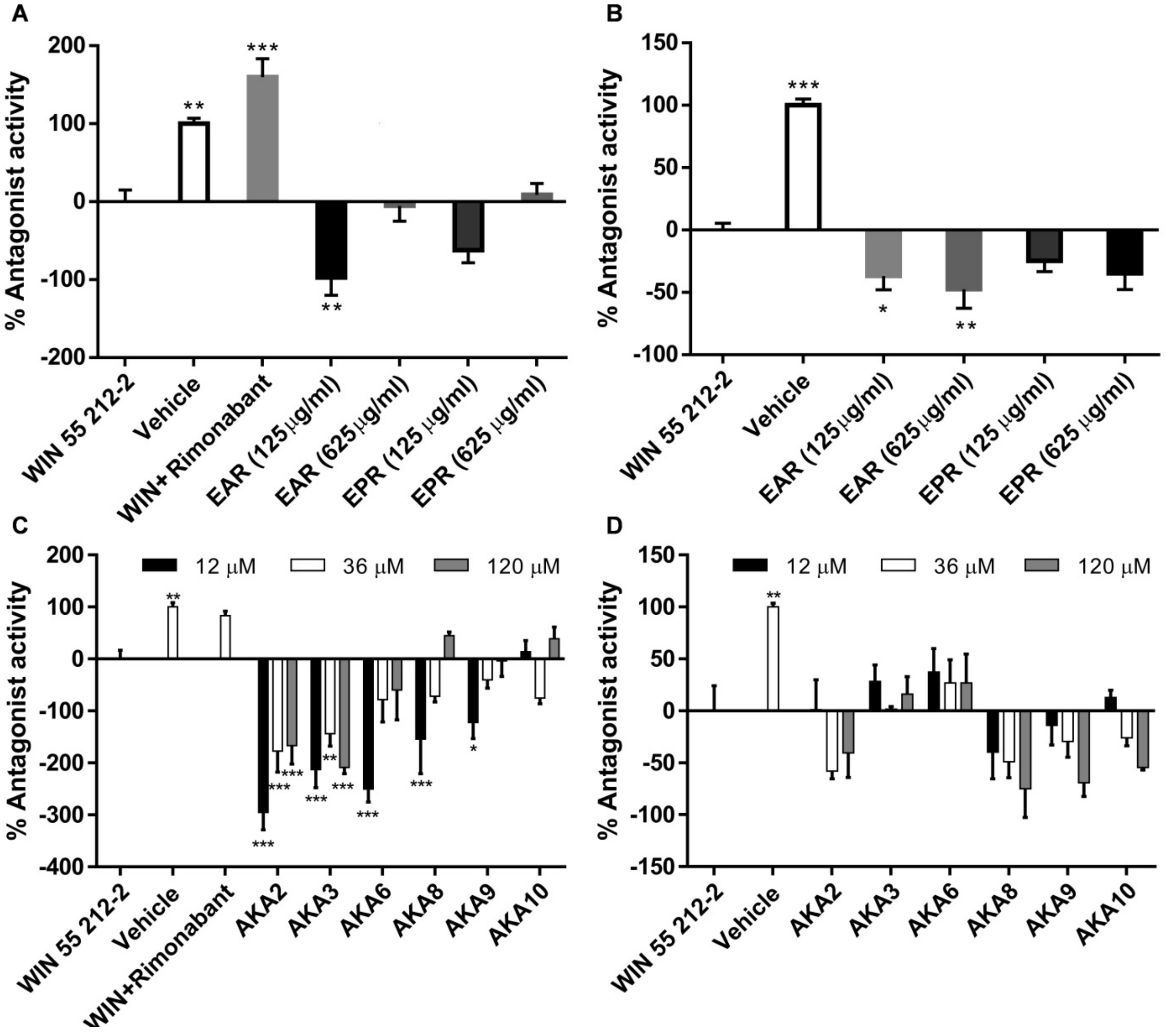

Supplemental figure - CB receptor antagonist effects. Mean antagonist effects of EAR (n=11) and EPR (n=9) extracts on CB1 (**A**) and CB2 (**B**) receptor internalization in the presence of agonist (WIN 55,212-2, 1 µM), expressed relative to agonist (with vehicle, 0% antagonist activity) and to the vehicle control (no CB activation, 100% antagonist activity). Rimonabant (SR141716, 10µM), the positive control, reversed agonist-induced internalization.. Mean antagonist effects of pure AKAs on CB1 (**C**) and CB2 (**D**) receptor in the presence of WIN 55,212-2 (1 µM). An analysis of variance (ANOVA) was performed to evaluate the variance between samples followed by Dunnett's test *post hoc* relative to the agonist (plus vehicle, 0.5 % ethanol) control. *: $p<0.05$; **: $p<0.01$.
